# Predicting cumulative live birth after IVF with growth hormone supplementation: the role of pretreatment duration and machine learning-based models

**DOI:** 10.3389/fendo.2026.1967517

**Published:** 2026-10-07

**Authors:** Yufeng Chen, Ziyi Dai, Peiyu Wang, Chang Liu

**Affiliations:** 1 Department of Gynaecology, The First People's Hospital of Jiashan, Jiashan Hospital Affiliated to Jiaxing University, Jiaxing, Zhejiang, China; 2 Department of Orthopedics, Wenzhou Hospital of Integrated Chinese and Western Medicine, Wenzhou, Zhejiang, China; 3 Reproductive Medicine Center, The First Affiliated Hospital of Wenzhou Medical University, Wenzhou, Zhejiang, China

**Keywords:** growth hormone, *in vitro* fertilization, live birth, machine learning, predictive models

## Abstract

**Aim:**

To investigate the association between growth hormone (GH) pretreatment duration and cumulative live birth (CLB), identify key prognostic factors, and develop predictive models using logistic regression (LR) and machine learning (ML) algorithms among women undergoing *in vitro* fertilization (IVF) with GH supplementation.

**Methods:**

We conducted a retrospective cohort study of women undergoing IVF/intracytoplasmic sperm injection (ICSI) with GH between February 2015 and June 2023 at a single tertiary center. Five models were developed: LR, random forest (RF), extreme gradient boosting (XGBoost), light gradient boosting machine (LightGBM), and support vector machine (SVM). Performance was assessed with cross-validation and bootstrap resampling. AUROC and Brier scores evaluated discrimination and calibration.

**Results:**

Among 1,782 women, 763 (42.82%) achieved at least one live birth. Maternal age, anti-Müllerian hormone (AMH), antral follicle count (AFC), the assisted reproductive technology (ART) cycle number, and GH duration were significant predictors. LR performed best (AUROC 0.782; Brier 0.187), showing strong calibration and interpretability. ML models had moderate discrimination but inferior calibration. AMH, AFC, and GH duration were key predictors across all methods.

**Conclusions:**

Both logistic regression and machine learning effectively predicted CLB after GH-pretreated IVF, but LR provided favorable discrimination, calibration, and interpretability. GH pretreatment ≥2 months was independently associated with higher odds of cumulative live birth.

## Introduction

*In vitro* fertilization (IVF) is a cornerstone of assisted reproductive technology (ART), offering hope to couples with infertility. Despite advances in protocols and technologies, success rates, particularly live birth rates, remain suboptimal and vary considerably among patients. This variability is influenced by ovarian reserve, response to controlled ovarian hyperstimulation, embryo quality, and endometrial factors.

Growth hormone (GH) has been increasingly used as an adjunct in IVF, especially for women with poor ovarian response, impaired embryo development, repeated implantation failure, or advanced age. While GH may improve oocyte and embryo quality, nearly half of IVF patients still fail to achieve pregnancy (1). The optimal timing and duration of GH supplementation remain unclear. Some patients may benefit from short-term administration at the start of gonadotropin (Gn) stimulation, whereas others may require prolonged pretreatment (2–4). This heterogeneity highlights the need for individualized treatment strategies.

Currently, decisions regarding GH use are largely empirical and not sufficiently guided by data-driven tools. Key factors such as female age, body mass index (BMI), anti-Müllerian hormone (AMH) levels, follicle-stimulating hormone (FSH) levels, antral follicle count (AFC), duration and type of infertility, and the timing of GH administration are known to influence IVF outcomes (5). It has been reported that obese women and women with advanced age could benefit more from GH treatment when experiencing IVF failure because of poor embryonic development (6). Nonetheless, the complex interplay of these factors, particularly in the context of GH pretreatment, remains poorly understood.

Predictive modeling provides a tool to integrate clinical and biochemical parameters for individualized outcome prediction (7–9). These models hold particular promise in addressing the complex and multifactorial nature of reproductive success, where single biomarkers often fail to provide sufficient predictive value. Recent studies have applied machine learning (ML) algorithms in IVF, taking advantage of their ability to capture nonlinear and high-dimensional interactions (10). However, many models do not consider GH duration, limiting their clinical utility (11–14). At the same time, traditional logistic regression (LR) remains widely used due to its interpretability and robustness in moderate-sized datasets, and in some cases may outperform more complex ML models prone to overfitting (15). Given these considerations, a direct comparison between traditional LR models and modern ML algorithms is warranted to determine the most reliable and clinically applicable approach. Therefore, in this study, we aimed to develop and compare predictive models for live birth following IVF in women treated with GH, using both logistic regression and several commonly used machine learning algorithms.

## Patients and methods

### Study design and population

This retrospective cohort study included women <40 years who underwent a GH-assisted IVF/intracytoplasmic sperm injection (ICSI) oocyte retrieval cycle at the Reproductive Medicine Center of the First Affiliated Hospital of Wenzhou Medical University between February 2015 and June 2023. Eligible patients received GH supplementation either prior to or during controlled ovarian hyperstimulation. GH was prescribed for patients with poor prognosis, including previous IVF failure, poor embryo quality, or reduced ovarian reserve, according to clinician judgement. The duration of GH supplementation was determined clinically and was not randomly assigned. Exclusion criteria were: (1) subsequent ART after achieving a live birth, (2) chromosomal abnormalities in either partner, (3) loss to follow-up ≥1 year, or (4) incomplete clinical or laboratory data. Clinical and laboratory parameters were extracted from electronic medical records. Each woman contributed only one GH-assisted oocyte retrieval cycle to the study. The study was approved by the Ethics Committees of the First Affiliated Hospital of Wenzhou Medical University (protocol number: KY2024-R226), with informed consent waived due to its retrospective nature. All procedures were conducted in accordance with the relevant guidelines and regulations.

### IVF procedure

All patients underwent standardized ovarian stimulation protocols, followed by oocyte retrieval, fertilization, and embryo transfer. Detailed descriptions of the stimulation protocols used at our center have been published previously (16). Recombinant human GH (Jintropin; GenSci, China) was administered at 4.5 IU every other day before controlled ovarian hyperstimulation and daily thereafter until oocyte retrieval. GH duration was defined as the interval from the initiation of GH supplementation to the day of oocyte retrieval and was categorized as <1 month, ≥1 to <2 months, ≥2 to <3 months, and ≥3 months. When the leading follicle reached 18 mm in diameter with at least three follicles >16 mm, 5,000–10,000 IU of hCG was administered for final oocyte maturation. Oocyte retrieval was performed 36 hours after the hCG trigger. No more than two embryos were transferred per cycle, in accordance with clinical guidelines. Luteal phase support was provided using oral dydrogesterone (20 mg/day) and vaginal progesterone (90 mg/day), starting on the day of oocyte retrieval and continuing for two weeks. If pregnancy was confirmed, luteal support was extended until the 8th week of gestation. Surplus embryos were cryopreserved by vitrification at either the cleavage stage (day 3) or blastocyst stage (day 5 or 6). For patients undergoing frozen embryo transfer (FET), endometrial preparation was performed using hormone replacement therapy, as previously described (20).

### Definitions

The primary outcome was cumulative live birth (CLB), defined as the occurrence of at least one live birth resulting from embryos generated from the GH-assisted IVF/ICSI oocyte retrieval cycle. CLB included live births following fresh embryo transfer and subsequent frozen-thawed embryo transfers using embryos derived from the same oocyte retrieval cycle, until a live birth was achieved or all available embryos from that cycle were exhausted (17). Singleton, twin, or higher-order deliveries were counted as one event. Patients with cycle cancellation due to no oocytes retrieved, no viable embryos, or other non-personal factors were classified as not achieving CLB. The ART cycle number was defined as the ordinal number of the IVF/ICSI oocyte retrieval cycle in which GH was administered. For example, an ART cycle number of 2 indicated that the GH-assisted oocyte retrieval was the woman’s second IVF/ICSI oocyte retrieval cycle. GH duration was determined from the actual duration of GH administration before oocyte retrieval.

### Statistical analysis and predictive modelling

Descriptive statistics were used to summarize baseline characteristics. Continuous variables were tested for normality and presented as mean ± standard deviation (SD) or median with interquartile range (IQR), as appropriate. Between-group comparisons were performed using Student’s t-test for normally distributed variables and the Mann-Whitney U test for non-normally distributed variables. Categorical variables were expressed as counts and percentages, and compared using the Chi-square test. Study subjects with missing clinical or laboratory data were excluded from the analysis, and no imputation was performed.

For predictive modelling, logistic regression (LR), random forest (RF), extreme gradient boosting (XGBoost), light gradient boosting machine (LightGBM), and support vector machine (SVM) were evaluated. Variables with *p* < 0.05 in multivariable logistic regression were considered significant predictors and were used to construct the LR-based models. For the machine learning models, two sets of predictors were evaluated: the five variables identified by logistic regression and the six variables with the highest importance according to the corresponding machine learning algorithms. Continuous variables without established clinical thresholds were categorized according to tertiles or clinically defined cutoffs, following the approach used in previous studies (18). The exact cutoff values for tertile-based categorization are provided in **Supplementary Table 2**. Internal validation was performed using tenfold cross-validation and 500 bootstrap resamples, in accordance with TRIPOD recommendations (19). Model discrimination was assessed by the area under the receiver operating characteristic curve (AUROC), and calibration was evaluated using Brier scores and calibration plots. Performance estimates and corresponding 95% confidence intervals were reported. No external validation was performed because this was a single-center study. Analyses were conducted using SPSS v27.0 and R v4.3.2. A two-tailed *p* < 0.05 was considered significant.

## Results

### General characteristics of study subjects

A total of 1,782 women were included after exclusions (**Supplementary Figure 1**). Among them, 763 achieved at least one live birth, corresponding to a cumulative live birth rate of 42.82%. The mean maternal age was 33.96 ± 3.82 years, and the mean infertility duration was 3.69 ± 3.03 years. Significant baseline differences between the live birth and non-live birth groups were observed in age of husband, maternal age, basal FSH, basal LH, AMH, AFC, ART cycle number, insemination method, Gn dosage, and GH duration, while maternal BMI, infertility duration, infertility type, basal estradiol (E2) levels, Gn duration, and embryo transfer type showed no significant difference (**Table 1**).

**Table 1:** General characteristics of study subjects.

Variables	Total (n=1782)	Live birth		*p* value
		Yes (n=763)	No (n=1019)	
Age of husband, years	35.87±4.70	35.25±4.67	36.33±4.67	<0.001
Maternal age, years	33.96±3.82	33.05±3.81	34.64±3.69	<0.001
Maternal BMI, kg/m2	21.82±3.10	21.82±3.01	21.82±3.17	0.99
Infertility duration, years	3.69±3.03	3.52±3.01	3.81±3.04	0.05
Infertility type (n, %)				0.66
Primary	554 (31.09)	233 (30.54)	321 (31.50)	
Secondary	1228 (68.91)	530 (69.46)	698 (68.50)	
Basal FSH, IU/L	8.23 (6.55-10.35)	7.76 (6.23-9.46)	8.68 (6.87-11.18)	<0.001
Basal E2, pmol/L	187.32±94.84	181.12±90.75	191.96±97.57	0.08
Basal LH, IU/L	4.00 (2.89-5.58)	4.14 (2.98-5.87)	3.94 (2.81-5.30)	<0.001
AMH, ng/mL	1.40 (0.79-2.53)	2.12 (1.21-3.93)	0.99 (0.59-1.71)	<0.001
AFC (n)	9.00 (6.00-13.00)	12.00 (8.00-17.00)	7.00 (5.00-10.00)	<0.001
ART cycle number (n)	2.00 (1.00-2.00)	2.00 (1.00-2.00)	2.00 (1.00-3.00)	<0.001
Insemination method (n, %)				<0.001
IVF	966 (54.21)	421 (55.18)	545 (53.48)	
ICSI	730 (40.96)	285 (37.35)	445 (43.67)	
IVF/ICSI	86 (4.83)	57 (7.47)	29 (2.85)	
Gn dosage, IU	2250.00 (1700.00-3075.00)	2025.00 (1575.00-2750.00)	2400.00 (1800.00-3425.00)	<0.001
Gn duration, days	10.00 (9.00-12.00)	10.00 (9.00-11.00)	10.00 (9.00-12.00)	0.07
GH duration, month				<0.001
< 1 month	1091 (61.22)	400 (52.42)	691 (67.81)	
≥1 to <2 months	216 (12.12)	94 (12.32)	122 (11.97)	
≥2 to <3 months	149 (8.36)	75 (9.83)	74 (7.26)	
≥ 3 months	326 (18.30)	194 (25.43)	132 (12.96)	
Embryo transfer (n, %)				0.44
Fresh ET cycle	373 (23.53)	173 (22.67)	200 (24.33)	
Froze ET cycle	1212 (76.47)	590 (77.33)	622 (75.67)	

BMI, body mass index; FSH, follicle-stimulating hormone; E2, estradiol; LH, luteinizing hormone; AMH, anti-Müllerian hormone; AFC, antral follicle count; ART, assisted reproductive technology; IVF, *in vitro* fertilization; ICSI, intracytoplasmic sperm injection; Gn, gonadotropin; ET, embryo transfer.

### Association between factors and live birth

To examine factors associated with cumulative live birth, maternal age was categorized based on established thresholds, and other quantitative variables were divided into tertiles. In univariate analysis, husband age, maternal age, AMH, AFC, ART cycle number, insemination method, Gn dosage, basal FSH, basal LH, and GH duration were significantly associated with live birth (**Table 2**). Women aged 36–39 years had lower odds of live birth compared with those ≤35 years (OR 0.51, p<0.001). Higher AMH and AFC levels were positively associated with live birth (AMH moderate vs low: OR 2.69, high vs low: OR 8.07; AFC moderate vs low: OR 3.12, high vs low: OR 8.93; all *p* < 0.001). GH treatment duration ≥2 to <3 months or ≥3 months was also associated with increased odds of live birth (OR 1.75, *p* = 0.001; OR 2.54, *p* < 0.001). In contrast, higher basal FSH, greater ART cycle number, and higher Gn dosage were inversely associated with live birth, while higher basal LH and IVF/ICSI insemination were positively associated.

**Table 2:** Univariate and multivariate logistic regression fitting results on associated factors of live birth.

Variables	Live birth (Yes)			
	Univariate model OR (90%CI)	*p* value	Multivariate model OR (95% CI)	*p* value
Age of husband (Ref: ≤35 years old)				
36-39 years old	0.71 (0.58-0.89)	0.002	1.15 (0.87-1.53)	0.33
≥40 years old	0.56 (0.44-0.72)	<0.001	1.00 (0.70-1.44)	0.99
Maternal age (Ref: ≤35 years old)				
36-39 years old	0.51 (0.41-0.63)	<0.001	0.73 (0.55-0.98)	0.04
Basal FSH (Ref: low level)				
Moderate level	0.78 (0.62-0.98)	0.03	0.97 (0.74-1.28)	0.83
High level	0.47 (0.37-0.59)	<0.001	0.98 (0.72-1.33)	0.90
Basal LH (Ref: low level)				
Moderate level	0.98 (0.77-1.23)	0.84	1.04 (0.79-1.38)	0.78
High level	1.34 (1.07-1.69)	0.01	1.32 (0.99-1.76)	0.06
AMH (Ref: low level)				
Moderate level	2.69 (2.08-3.48)	<0.001	1.77 (1.33-2.37)	<0.001
High level	8.07 (6.20-10.50)	<0.001	2.84 (2.03-3.98)	<0.001
AFC (Ref: low level)				
Moderate level	3.12 (2.41-4.04)	<0.001	2.05 (1.53-2.75)	<0.001
High level	8.93 (6.89-11.58)	<0.001	3.42 (2.45-4.77)	<0.001
ART cycle number (+1)	0.84 (0.78-0.91)	<0.001	0.82 (0.73-0.92)	<0.001
Insemination method (Ref: IVF)				
ICSI	0.83 (0.68-1.01)	0.06	0.94 (0.74-1.20)	0.63
IVF/ICSI	2.54 (1.60-4.05)	<0.001	1.64 (0.96-2.81)	0.07
Gn dosage (Ref: low level)				
Moderate level	0.71 (0.56-0.89)	0.003	0.94 (0.72-1.24)	0.67
High level	0.45 (0.35-0.57)	<0.001	0.82 (0.61-1.10)	0.19
GH duration (Ref: <1 month)				
≥1 to <2 months	1.33 (0.99-1.79)	0.06	1.37 (0.97-1.95)	0.07
≥2 to <3 months	1.75 (1.24-2.47)	0.001	1.68 (1.11-2.56)	0.02
≥ 3 months	2.54 (1.97-3.27)	<0.001	1.75 (1.28-2.38)	<0.001

Ref, reference; FSH, follicle-stimulating hormone; LH, luteinizing hormone; AMH, anti-Müllerian hormone; AFC, antral follicle count; ART, assisted reproductive technology; IVF, *in vitro* fertilization; ICSI, intracytoplasmic sperm injection; Gn, gonadotropin; GH, growth hormone; OR, odds ratio; CI, confidence interval.

Variables with *p* < 0.05 in univariate analysis were entered into a multivariate logistic regression model. Maternal age, AMH, AFC, GH treatment duration, and ART cycle number remained independent predictors of cumulative live birth. Specifically, women aged 36–39 years had lower odds of live birth than those ≤35 years (OR 0.73, *p* = 0.04), while high AMH and AFC levels were associated with increased odds (OR 2.84 and 3.42, both *p* < 0.001). GH treatment duration ≥2 to <3 months and ≥3 months remained positively associated (OR 1.68 and 1.75, both *p* < 0.05), whereas the ART cycle number continued to show an inverse relationship with live birth (OR 0.82, *p* < 0.001).

### Variable importance across machine learning models

Variable importance for predicting cumulative live birth was assessed using four machine learning algorithms: Random Forest (RF), XGBoost, LightGBM, and Support Vector Machine (SVM) (**Figure 1**). Across most models, AMH and AFC consistently ranked among the top predictors. LightGBM additionally prioritized LH and FSH, while SVM highlighted maternal age and GH treatment duration. Gn dosage showed moderate importance in RF, XGBoost, and LightGBM, but less influence in SVM. Insemination method was consistently the least important predictor across all models.

**Figure 1:**
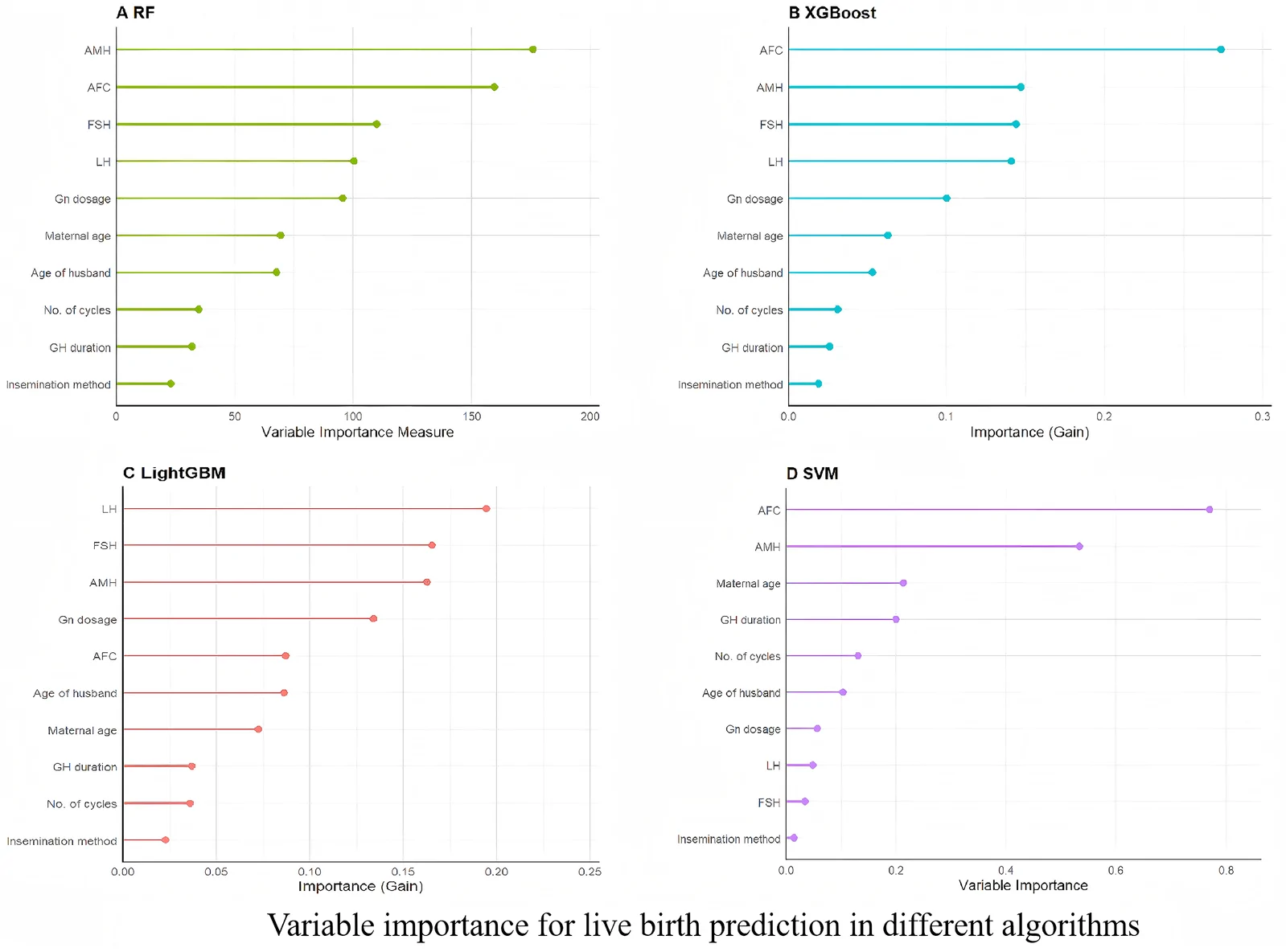
Variable importance for live birth prediction in different algorithms. **(A)** Random forest (RF); **(B)** extreme gradient boosting (XGBoost); **(C)** light gradient boosting machine (LightGBM); **(D)** support vector machine (SVM). Variable importance measures are algorithm-specific and should not be interpreted as directly comparable across algorithms. AMH, anti-Müllerian hormone; AFC, antral follicle count; FSH, follicle-stimulating hormone; LH, luteinizing hormone; Gn, gonadotropin; GH, growth hormone.

### Model performance evaluation

The predictive performance of logistic regression and four machine learning algorithms (RF, XGBoost, LightGBM, and SVM) was internally validated using both cross-validation and bootstrap resampling, with AUROC and Brier score as the main evaluation metrics (**Figure 2**). Overall, all models demonstrated acceptable discrimination and calibration. Logistic regression using the five regression-derived variables achieved the best overall balance between discrimination and calibration, with a cross-validated AUROC of approximately 0.78 and a Brier score below 0.19. Among the machine learning algorithms, models constructed with the five regression-derived variables consistently outperformed those using the top six ML-selected variables. Random forest, XGBoost, and LightGBM all showed modest improvements in AUROC (typically 0.02-0.03 higher) and slightly lower Brier scores when using the regression-derived variable set. The SVM model demonstrated stable performance across both validation strategies and variable selections, with AUROC around 0.75-0.77 and Brier scores near 0.20. Detailed performance metrics with 95% confidence intervals for each algorithm and validation approach are presented in **Supplementary Table 1**. Moreover, the calibration plots for all five models provided in **Supplementary Figure 2** showed that the logistic regression exhibited the most consistent alignment with the perfect calibration line.

**Figure 2:**
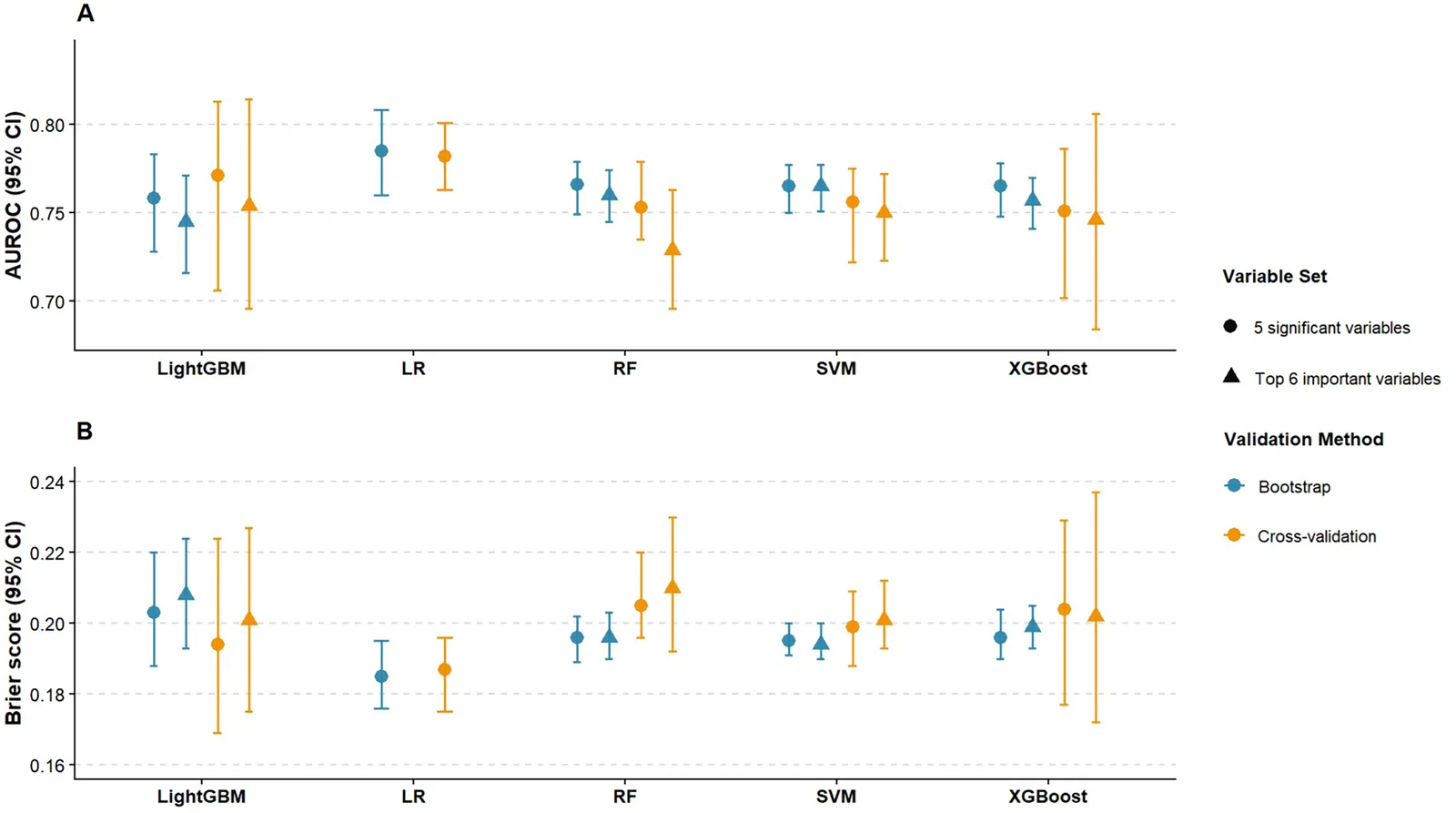
Comparative internal validation of different machine learning algorithms for live birth prediction. **(A)** Comparison of the discriminative ability (AUROC with 95% CI) of different machine learning algorithms, including logistic regression (LR), random forest (RF), extreme gradient boosting (XGBoost), light gradient boosting machine (LightGBM), and support vector machine (SVM). **(B)** Comparison of the calibration performance (Brier score with 95% CI) of the same algorithms. Both cross-validation and bootstrap methods were used for internal validation, and models were built based on either the five significant variables or the top six important variables.

## Discussion

This study compared LR with four ML algorithms for predicting cumulative live birth in women undergoing IVF with GH treatment. The LR model demonstrated strong discriminatory ability and good calibration, while ML models showed competitive performance but were less well-calibrated. Key predictors such as AMH, AFC, and GH treatment duration were consistently identified across models, highlighting their critical roles in IVF outcome prediction.

The variables identified as key predictors of cumulative live birth in our study were largely consistent with those found in previous studies on IVF outcomes (20–22). Maternal age, AMH, AFC, and the ART cycle number were all significantly associated with live birth rates. Specifically, maternal age of 36–39 years was associated with lower odds of live birth, in line with the well-documented age-related decline in fertility (23). High AMH and AFC levels were positively correlated with better IVF outcomes, reinforcing their role as reliable markers of ovarian reserve and follicular response (24). Moreover, a higher ART cycle number was inversely associated with cumulative live birth. This finding may reflect the clinical characteristics and treatment history of women who reached a later GH-assisted IVF/ICSI cycle rather than a direct effect of repeated treatment itself (21).

Our study showed that both LR and ML models offered insights into predictors of cumulative live birth, but their performance differed. LR consistently outperformed ML algorithms, achieving an AUROC of 0.782 with excellent calibration, while ML models such as RF (AUROC 0.729) demonstrated only moderate discrimination and poorer calibration, highlighting challenges of stability and generalizability in IVF datasets. Moreover, SVM showed robustness with smaller datasets but struggled with calibration and feature selection, which affected overall predictive accuracy (25). Gradient Boosting models such as XGBoost and LightGBM could capture nonlinear relationships but required parameter tuning and risked overfitting. Even with the five key variables identified by LR (AMH, AFC, age, ART cycle number, GH duration), ML models did not exceed LR in AUROC or Brier score. This suggests that while ML can model complex interactions, it may face calibration and generalization challenges in smaller IVF datasets. Still, ML highlighted variable importance, with AMH and AFC consistently ranked as the strongest predictors.

The superior performance of LR in our study can be attributed to its simpler structure and fewer parameters, making it more resilient to overfitting in small clinical datasets. This aligns with findings from other research, where logistic regression was shown to provide robust, clinically interpretable predictions, especially when datasets are limited. Additionally, the clear interpretability of LR allows clinicians to easily understand the relationships between predictors and the predicted outcomes, an essential feature for clinical adoption.

A recent study involving 48,514 fresh IVF cycles also explored machine learning models for predicting live birth outcomes and identified key predictors such as maternal age and AFC, which are consistent with our findings (11). However, they did not compare ML with logistic regression, limiting insights into performance relative to traditional approaches. For large datasets, ML may offer greater flexibility and predictive power by capturing complex nonlinear relationships (15). Another study developed a mathematical model using age, AMH, and ART cycle number to predict live birth, similarly highlighting these variables as crucial predictors (26). Similar to our findings, their study demonstrated that age, AMH and the ART cycle number are crucial predictors of live birth outcomes. While they focused on age-related changes in oocyte quality and quantity, our study highlights the utility of these same variables in the context of GH pretreatment, showing how different factors may contribute to IVF success.

The inclusion of GH treatment duration in our predictive model is of clinical interest because follicle development from the primordial stage to pre-ovulatory maturity typically takes about 85 days (27). The biological effects of GH on ovarian function may involve multiple processes related to oocyte maturation, including energy metabolism, mitochondrial function, intracellular signaling, and apoptosis regulation (28, 29). Some studies have reported improvements in ovarian response, embryo-related outcomes, or live birth, whereas others have not demonstrated consistent benefits (3, 6, 30, 31). The optimal timing and duration of GH supplementation also remain uncertain. Previous research has indicated that short GH pre-treatment durations (< 2 months) do not significantly increase live birth rates (32–35). In a recent retrospective cohort study of women with poor embryo quality in previous IVF cycles, 12 weeks of GH pretreatment was associated with a higher D3 good-quality embryo rate and higher blastocyst formation rate (36). These findings suggest that prolonged GH exposure may be associated with better embryo-related outcomes. In our cohort, GH pretreatment for ≥2 months was associated with a higher likelihood of cumulative live birth after adjustment for other clinical predictors. However, this association should be interpreted as a prognostic association among women who received GH rather than evidence of a causal benefit from prolonged GH supplementation.

A key feature of our study was the incorporation of GH treatment duration into the prediction of cumulative live birth. GH use in IVF is primarily guided by clinical observations suggesting benefits for women with poor ovarian response, low ovarian reserve, advanced age, or repeated implantation failure (37, 38). Its use was guided by individualized clinical judgment rather than standardized protocols, based on patient history and evidence from smaller studies in specific subgroups (39). This clinical heterogeneity may contribute to differences in outcomes across treatment durations. Therefore, GH duration should be regarded as a prognostic variable within the GH-treated population rather than as a measure of treatment efficacy. The predictive value of GH duration may help characterize prognosis among women receiving GH, but whether a specific duration provides a causal reproductive benefit remains to be established.

Despite the strengths of our findings, several limitations must be considered. First, the study sample was collected from a single medical institution using a retrospective design, which inevitably introduced the potential for data and selection biases. Second, although the predictive models we tested provided valuable insights into the factors influencing live birth, their generalizability should be confirmed through validation in larger, external datasets. Third, other potential biomarkers and clinical variables, such as the endometrial environment, lifestyle factors, and comorbidities, may further refine the predictive accuracy of these models. Fourth, several continuous variables were categorized into tertiles for model construction. Although this approach was based on previous studies, categorization may result in some loss of information, and sensitivity analyses using continuous or nonlinear terms were not performed. Therefore, potential nonlinear associations between continuous predictors and cumulative live birth cannot be fully excluded. Moreover, GH duration was determined clinically rather than randomly assigned. It may therefore reflect underlying prognosis, treatment indication, clinician preference, or other clinical considerations. Because the study included only women who received GH, the observed association between GH duration and cumulative live birth should not be interpreted as evidence of a treatment effect. Residual confounding and confounding by indication cannot be excluded. Prospective randomized studies are needed to establish whether the duration of GH supplementation has a causal effect on cumulative live birth. Future research should also evaluate the performance of these models in external cohorts and assess their integration into clinical decision-making.

In conclusion, both logistic regression and machine learning algorithms show potential for predicting cumulative live birth in IVF patients treated with GH. LR demonstrated favorable discrimination, calibration, and interpretability. Notably, GH pretreatment for ≥2 months was independently associated with higher odds of cumulative live birth in this cohort, although further studies with appropriate control groups are required to confirm this association.

## Data Availability

The raw data supporting the conclusions of this article will be made available by the authors, without undue reservation.
