# Supplementary material for: Predicting cumulative live birth after IVF with growth hormone supplementation: the role of pretreatment duration and machine learning-based models
Source: Front Endocrinol (Lausanne). 2026 Oct 7;17:1967517. doi: 10.3389/fendo.2026.1967517 (PMC13682970; doi:10.3389/fendo.2026.1967517)
Supplement: Supplementary file 1 [file Table1.docx]

Supplementary Table 1. Performance of different machine learning algorithms by using internal validation

| Algorithms | Variable selected | Cross-validation | | Bootstrap | |
| --- | --- | --- | --- | --- | --- |
|  |  | AUROC (95% CI) | Brier score (95% CI) | AUROC (95% CI) | Brier score (95% CI) |
| LR | 5 significant variables | 0.782 (0.763-0.801) | 0.187 (0.175-0.196) | 0.785 (0.760-0.808) | 0.185 (0.176-0.195) |
| RF | Top 6 important variables | 0.729 (0.696-0.763 ) | 0.210 (0.192-0.230) | 0.760 (0.745-0.774) | 0.196 (0.190-0.203) |
|  | 5 significant variables | 0.753 (0.735-0.779) | 0.205 (0.196-0.220) | 0.766 (0.749-0.779) | 0.196 (0.189-0.202) |
| XG Boost | Top 6 important variables | 0.746 (0.684-0.806) | 0.202 (0.172-0.237) | 0.757 (0.741-0.770) | 0.199 (0.193-0.205) |
|  | 5 significant variables | 0.751 (0.702-0.786) | 0.204 (0.177-0.229) | 0.765 (0.748-0.778) | 0.196 (0.190-0.204) |
| Light GBM | Top 6 important variables | 0.754 (0.696-0.814) | 0.201 (0.175-0.227) | 0.745 (0.716-0.771) | 0.208 (0.193-0.224) |
|  | 5 significant variables | 0.771 (0.706-0.813) | 0.194 (0.169-0.224) | 0.758 (0.728-0.783) | 0.203 (0.188-0.220) |
| SVM | Top 6 important variables | 0.750 (0.723-0.772) | 0.201 (0.193-0.212) | 0.765 (0.751-0.777) | 0.194 (0.190-0.200) |
|  | 5 significant variables | 0.756 (0.722-0.775) | 0.199 (0.188-0.209) | 0.765 (0.750-0.777) | 0.195 (0.191-0.200) |
