# Supplementary material for: Predicting cumulative live birth after IVF with growth hormone supplementation: the role of pretreatment duration and machine learning-based models
Source: Front Endocrinol (Lausanne). 2026 Oct 7;17:1967517. doi: 10.3389/fendo.2026.1967517 (PMC13682970; doi:10.3389/fendo.2026.1967517)
Supplement: Supplementary file 2 [file Table2.docx]

Supplementary Table 2. Cutoff values used for categorization of continuous variables

| Variables | First tertile | Second tertile |
| --- | --- | --- |
| Basal FSH, IU/L | 7.10 | 9.40 |
| Basal LH, IU/L | 3.32 | 4.99 |
| AMH, ng/mL | 0.93 | 2.14 |
| AFC (n) | 7 | 12 |
| Gn dosage, IU | 1800 | 2725 |

Note: Continuous variables without established clinical thresholds were categorized into tertiles based on the corresponding cutoff values shown above.
